# A Robust and Real-Time Capable Envelope-Based Algorithm for Heart Sound Classification: Validation under Different Physiological Conditions

**DOI:** 10.3390/s20040972

**Published:** 2020-02-11

**Authors:** Angelika Thalmayer, Samuel Zeising, Georg Fischer, Jens Kirchner

**Affiliations:** Institute for Electronics Engineering, Friedrich-Alexander-Universität Erlangen-Nürnberg (FAU), 91058 Erlangen, Germany; georg.fischer@fau.de (G.F.); jens.kirchner@fau.de (J.K.)

**Keywords:** heart sounds, envelope, hilbert transform, short-time fourier transform, classification, real-time, auscultation, robust

## Abstract

This paper proposes a robust and real-time capable algorithm for classification of the first and second heart sounds. The classification algorithm is based on the evaluation of the envelope curve of the phonocardiogram. For the evaluation, in contrast to other studies, measurements on 12 probands were conducted in different physiological conditions. Moreover, for each measurement the auscultation point, posture and physical stress were varied. The proposed envelope-based algorithm is tested with two different methods for envelope curve extraction: the Hilbert transform and the short-time Fourier transform. The performance of the classification of the first heart sounds is evaluated by using a reference electrocardiogram. Overall, by using the Hilbert transform, the algorithm has a better performance regarding the F_1_-score and computational effort. The proposed algorithm achieves for the S_1_ classification an F_1_-score up to 95.7% and in average 90.5%. The algorithm is robust against the age, BMI, posture, heart rate and auscultation point (except measurements on the back) of the subjects.

## 1. Introduction

Cardiovascular diseases are the leading cause of death worldwide [1,2]. According to [3], this trend will continue and deteriorate in the future. Apart from the personal consequences, the healthcare costs are a huge burden for society [3].

One approach to tackle this problem is the daily monitoring of vital parameters, e.g., from the electrocardiogram (ECG) and phonocardiogram (PCG) (Supplementary Materials) by use of wearable sensors [4], since abnormal properties can indicate cardiac diseases. For the latter type of signals, automatic heart sound detection and classification algorithms are under research. The most successful approaches are the envelope-based, probabilistic-based and the feature-based methods [5].

Feature-based methods, as e.g., proposed by [6,7,8,9,10], extract features such as the Shannon entropy, discrete wavelet transform (DWT), continuous wavelet transform (CWT) or mel-frequency cepstral coefficients (MFCC) out of the PCG signal. With the use of classifiers (for example support vector machine (SVM), twin support vector machine (TWSVM) or deep neural networks (DNN)), it can be determined, if the features correspond to a heart sound [5]. However, feature-based methods have the disadvantages of high computational effort and strong dependency on datasets for training [5]. As the PCG strongly varies between the subjects as well as with the posture, heart rate and auscultation point, this would require separate data sets for each of these conditions.

Probabilistic-based methods classify heart sounds by different features, e.g., time-frequency energy or average distance between two peaks. The hidden-Markov model (HMM) is a well-established probabilistic-based model for the classification of heart sounds [11]. In [12,13] the HMM, which Schmidt et al. proposed for the heart sound classification, was improved to the hidden-semi-Markov model (HSMM) and tested with a huge amount of test persons. Renna et al. used convolutional neural networks (CNN) together with an underlying HMM in order to outperform the state of the art on heart sound classification [14]. However, the probabilistic-based methods also have a strong dependency on datasets for training and the computational effort is similar to that of the feature-based methods.

Envelope-based approaches, in contrast, are characterized by low computational costs and promise applicability under various situations in the daily life of the subjects without a dedicated training for these conditions. This robustness is of particular importance, as some pathological behaviour can only be observed during physical effort [15]. Nevertheless, the influence of the posture, auscultation point, physical stress and breathing on heart sound classification has not been addressed so far, to the best of the authors’ knowledge.

Typical methods used for the extraction of the envelope curve are the normalized average Shannon energy [16,17] or Hilbert transform (respectively short-time modified Hilbert transform (STMHT)) [18]. For the classification of the heart sounds, the peaks in the envelope curve are detected. The distances between two consecutive peaks are segmented in systoles and diastoles. Therefore, the first heart sound must be located at the beginning of the systole, whereas the second heart sound at the beginning of the diastole. The heart sound classification of the envelope-based algorithms use the assumption that the systole is shorter than the diastole [5,19]. However, at increased heart rates, the systole and diastole period are roughly equal in time, which can lead to errors in the detection of heart sounds [19,20]. Moreover, additional peaks e.g., noise, split heart sounds, third and forth heart sounds are problematic for envelope-based approaches [5,21].

In this paper, an enhanced envelope-based algorithm for automatic real-time detection and classification of the first and second heart sound is presented. The proposed algorithm distinguishes between increased (>80 bps) and normal heart rates, and in this way deals with the aforementioned problem that for increased heart rates, the length of the systole and diastole are almost equal. Furthermore, a real-time capable algorithm is needed for daily monitoring of heart sounds. In the literature, the autocorrelation (ACF) is well-established for heart rate estimation out of PCG signals [11,13,20,22]. In 2019, Dia et al. proposed a method for extracting the heart rate from noisy PCG signals by using the non-negative matrix factorization (NMF) [23]. They applied the NMF on the spectrogram of a PCG in order to estimate the heart rate. In the proposed paper an approach is made to keep the overall computational effort as low as possible. This is accomplished by using the ACF for heart rate estimation and an envelope-based classification algorithm.

For evaluating the proposed algorithm, measurements of the ECG and PCG were conducted on twelve male subjects with different age and body-mass index (BMI). The posture, the auscultation point and the physical stress were varied in order to evaluate the robustness of the presented algorithm in daily routine activities of a subject. Finally, the algorithm for heart sound classification was tested and evaluated with two different methods for envelope curve extraction, namely the Hilbert transform (HT) and short-time Fourier transform (STFT) and their corresponding results were compared.

This paper is organized as follows. Section 2 explains the fundamentals of the heart cycle and the applied signal processing methods of the algorithm. In Section 3, the developed algorithm for heart sound classification is introduced. The algorithm is tested with two different approaches for envelope curve extraction, namely the HT and the STFT. The proposed parameters for evaluating and optimization the performance of the algorithm are presented in Section 4. The process of the data acquisition, the study population and the used hardware setup is introduced in Section 5. The results for the classification process for the two different approaches are evaluated, compared and discussed in Section 6. Finally, in Section 7 the findings are concluded.

## 2. Fundamentals

### 2.1. Heart Cycle and Heart Sounds

**Figure 1:**
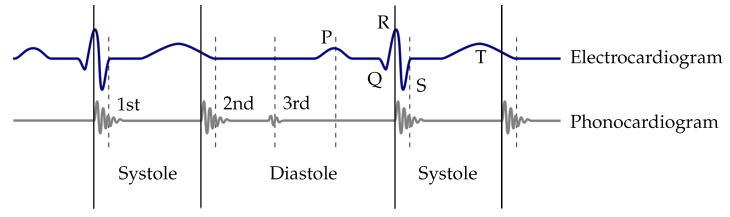
Wiggers diagram: the blue curve shows the ECG and the grey curve the related heart sounds (PCG). A heart cycle is defined as a sequence of a systole and a diastole [27].

The heart cycle consists of the systole and diastole, which correspond to the contraction and relaxation of the heart, respectively. The beginning of the systole is marked by the beginning of the first heart sound S_1_, the diastole starts with the second heart sound S_2_. Compared to the ECG, the beginning of S_1_ is synchronous to the peak of the R-wave (Figure 1). Within the diastole, the third S_3_ and fourth S_4_ heart sound occur. However, the third and fourth heart sound are only heard occasionally during an auscultation [24]. Since S_4_ has less diagnostic value, it is neglected in Figure 1 [25]. The frequency spectrum of heart sounds is approximately 20–200 Hz [21,26]. The ratio of the systole and the diastole is 1:2 in the resting heart rate and decreases with higher heart rates. The optimal auscultation point is called Erb’s point. At this point the heart sounds have the highest amplitudes and, therefore, the best results for auscultation can be achieved. [27]

### 2.2. Methods for Envelope Curve Extraction

The PCG is a periodical signal, however it has a non-linear and non-stationary characteristic. In consequence, the frequency changes over time. For the detection of the heart sounds, the PCG is transformed to a simpler signal to investigate the intrinsic characteristic.

#### 2.2.1. Hilbert Transform (HT)

The envelope curve of the PCG can be extracted with the HT. This method is appropriate for narrowband signals like heart sounds and can be computed with low computational costs [28]. The HT of a real-valued and time-continuous signal x(t) is defined as [29,30]

(1) $$H\left(x(t)\right)=\frac{1}{\pi }\int_{-\infty }^{\infty }\frac{x(\tau )}{t-\tau }\;d\tau .$$

The related envelope curve EH(x(t)) of x(t) is computed with

(2) $$E_{H}\left(x\left(t\right)\right)=\sqrt{x{\left(t\right)}^{2}+H{\left(x(t)\right)}^{2}}.$$

#### 2.2.2. Short-Time Fourier Transform (STFT)

The Fourier transform (FT) extracts the spectral components of a signal. However, if the signal is non-stationary, it is not possible to reconstruct the signal in the time domain, since the coherence of time and frequency is lost. Thus, the STFT is introduced, which solves this problem by computing the FT only within a limited time window of length *b*. This includes the assumption that the signal is stationary during this time window [31]. The window is shifted along the time axis, with an overlap between the windows, which is denoted as *k* and given in percent. To restrict the full signal x(t) to the window interval, x(t) is multiplied by a window function w(τ). The STFT is computed as proposed in [32]

(3) $$S\left(t,\omega \right)=\int_{-\infty }^{\infty }x\left(\tau \right)w\left(\tau -t\right)e^{-j\omega \tau }d\tau .$$

Rabiner et al. figured out that a Hamming window fits best for non-stationary audio signals (like PCGs) [33]. The choice of the window length and the overlap are essential, since the time- and frequency resolution depend on it. A short window is related to a high time and low frequency resolution, whereas a long window results in a low time and a high frequency resolution. Therefore, *k* and *b* are optimized for the heart sound classification.

For detecting the heart sounds, the envelope curve is extracted by computing the power-spectral- density *P*, which is also called the spectrogram. It is derived out of the Fourier coefficients [31]:

(4) $$P\left(S\left(t,\omega \right)\right)={\left|S\left(t,\omega \right)\right|}^{2}$$

For extracting the envelope curve, the maximum of *P* is determined for each time-step.

## 3. Algorithm for Heart Sound Classification

**Figure 2:**
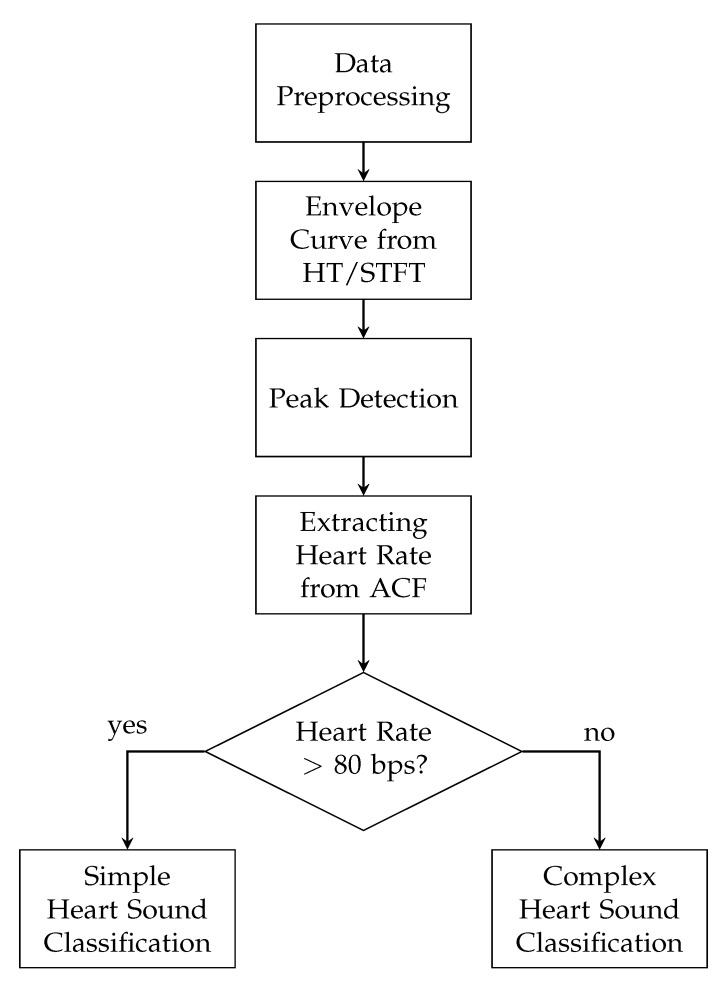
Flowchart of the proposed algorithm for the detection and classification of heart sounds.

The classification algorithm was implemented in MATLABmathsizesmall R2019a. The scheme of the algorithm for the detection and classification of heart sounds is illustrated in Figure 2. The algorithm sequence started with the data preprocessing, which includes filtering the raw-data and synchronizing the PCG and ECG signal. An initial segment of the PCG signals (60 s) of 8s length, which was used for synchronization (see. Section 3.1.2), was discarded. The remaining PCG signals were divided into five intervals of equal duration and further analysed separately. In the next step, the envelope curve was extracted from the filtered PCG signal for both, the HT and STFT. For both envelope curves, the algorithm was applied separately. The peaks of the envelope curve were detected and the heart rate was estimated by using the autocorrelation function (ACF). In this way, the proposed algorithm distinguished between increased (>80 bps) and normal heart rates and classified the heart sounds, using two different approaches. The single steps of the algorithm are explained in the following.

### 3.1. Data Preprocessing

#### 3.1.1. Filtering of the Raw-Data

**Figure 3:**
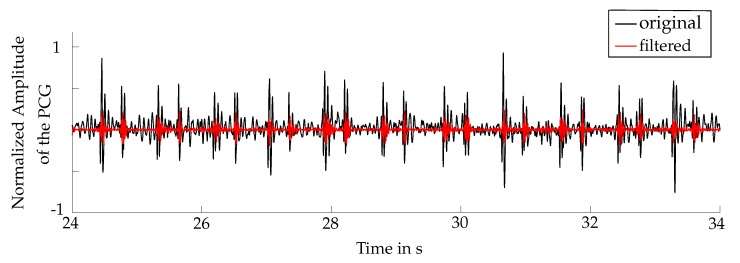
Comparison between the original phonocardiogram (PCG) in black and the bandpass filtered PCG in red.

For eliminating the noise, which is caused by respiration, human speech, lung sounds or movement of the stethoscope, the PCG was filtered. The applied filter was a Butterworth bandpass, consisting of a low-pass and a high-pass filter, with a passband from $f_{\mathrm{lower}}$ to $f_{\mathrm{upper}}$ and filter orders $N_{\mathrm{LP}}$ and $N_{\mathrm{HP}}$, respectively. Those parameters were used for the optimization of the algorithm (see Section 6.1). In Figure 3 a comparison between the raw data and the filtered PCG is illustrated.

#### 3.1.2. Synchronization of the PCG and ECG Signal

As the R-peaks in the ECG are simultaneous with the first heart sounds S_1_ (see Figure 1), the R-peaks were used to evaluate the correct detection and classification of S_1_. For that purpose, the PCG and ECG were synchronised by use of artefacts induced by knocking three times on the electrodes of the ECG. Therefore, the first 8 s of the PCG signals were excluded.

### 3.2. Envelope Curves

**Figure 4:**
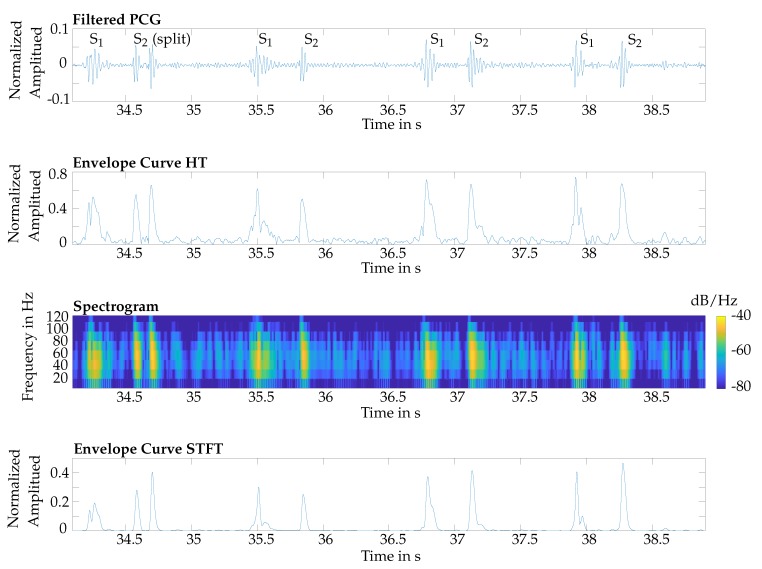
Top: filtered PCG of a reference measurement; second: related envelope curve derived by the HT; third: spectrogram of the short-time Fourier transform (STFT); bottom: envelope curve derived by the STFT. Note: the first S_2_ is split.

The basis of the algorithm was the envelope curve of the PCG, which was extracted by two different methods, namely the HT and the STFT (Figure 4). The first subplot shows a filtered PCG signal and the corresponding envelope curve, derived by the HT, is shown in the second subplot. In the third subplot, the spectral power in dB/Hz is plotted over time and frequency. The heart sounds have a high power density, so for detecting S_1_ and S_2_ the maximum value of the power spectral density is computed for every time step. These values are shown in the fourth subplot of Figure 4. The resulting curve was similar to the envelope curve derived by the HT.

### 3.3. Peak Detection

The detection of the peaks in the envelope curve was realized by computing the gradient. The conditions for a local maximum are a changing sign of the gradient from positive to negative. All peaks with an amplitude, which was larger than the defined threshold were considered for the heart sound classification:

(5) $$x_{\mathrm{env}}\left(t\right)>\mathrm{mean}\left[x_{\mathrm{env}}\left(t\right)\right]\cdot n$$

where $x_{\mathrm{env}}(t)$ is the envelope curve and *n* is an arbitrary parameter, which is optimized (see Section 6.1).

Furthermore, it was essential to restrict two maxima within the length of a heart sound. Therefore, a time window of 150 ms, which approximately corresponded to the maximal length of a heart sound [5,34], was applied. The global maximum within the window was assigned as the detected maximum and, therefore, as a potential heart sound. In consequence, the algorithm was able to deal with split heart sounds.

### 3.4. Extracting the Heart Rate

**Figure 5:**
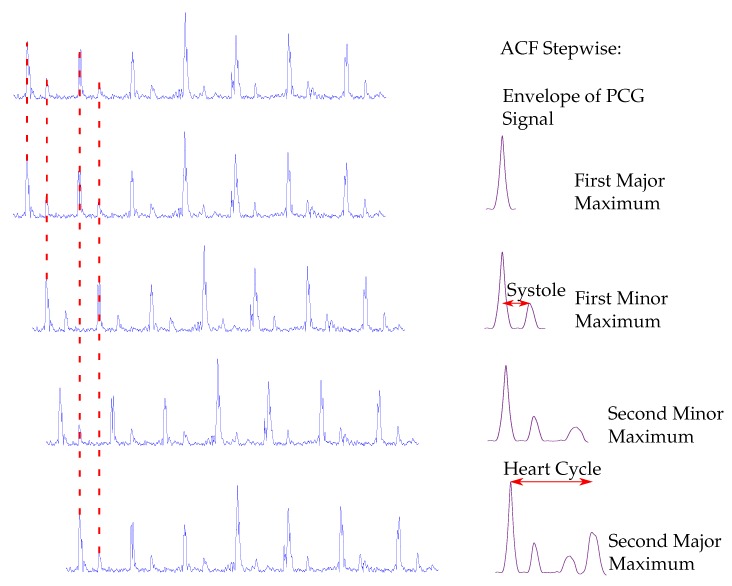
Procedure to determine the heart cycle and the systole length from the autocorrelation (ACF). On the left side the envelope of the PCG is shown. In the respective rows, it is shifted in time. For reasons of clarity and comprehensibility, dashed lines are not drawn for all heart sounds. On the right side, the corresponding ACF is shown.

As shown in Figure 5 the heart cycle, as well as the length of the systole, can be computed by using the ACF, which is a robust and well-established tool for the heart rate estimation. Thus, the local maxima of the ACF have to be extracted. The PCG is a quasi periodic signal and has finite length, therefore, the local maxima of the ACF are periodic with decaying amplitudes, since the signal is shifted and in consequence the overlapping of the signals gets smaller.

The first major maximum occurs when the original signal and the shifted signal fully overlap. At the first minor maximum, the second heart sounds of the origin signal and the first heart sounds of the shifted signal are overlapping, whereas the second minor maximum appears, when the first heart sounds of the original signal and the second heart sounds of the shifted signal interfere. The second major maximum appears, when the shifted signal again fully overlaps with the original signal. Thus, the average heart cycle corresponds to the distance between the first two major maxima. The distance between the first major maximum and the first minor maximum is extracted and corresponds to the average length of the systole (SYS). The second major maximum is extracted by a global maximum search within an interval of 1.5 s after the first major maximum. This corresponds to a heart rate of 40 bps, which is chosen as lower boundary of the heart rate for the proposed algorithm. In [35,36] an alternative approach for estimating the systolic length is presented. It is stated that the length of a systole decreases linear with the heart rate (HR). Therefore, the extracted HR of the ACF can be used to estimate the length of a systole in ms according to the empirical formulas

(6) $$\mathrm{SYS}=\left\{\begin{array}{cc} -1.14\;\mathrm{HR}+371.55\;\mathrm{ms} & \mathrm{if}\;\mathrm{HR}>80\;\mathrm{bps}\\ -6.58\;\mathrm{HR}+766.44\;\mathrm{ms} & \mathrm{otherwise}. \end{array}\right.$$

The two methods for the estimation of the systolic length were both tested for the heart sound classification. The results of the comparison are presented in Section 6.2. The average HR was the reciprocal value of the average heart cycle. The average diastole length (DIA) in ms is computed with

(7) $$\mathrm{DIA}=\frac{60\times 1000}{\mathrm{HR}}-\mathrm{SYS}.$$

The length of a systole is defined as the distance between the beginning of S_1_ and the beginning of S_2_ (see Section 2). However, for the algorithm, the length of a systole is determined by the distance between the major peaks of S_1_ and S_2_. Heart sounds can be split especially during inhalation [37,38,39] (like first S_1_ in Figure 10, or first S_2_ in Figure 4). In the peak detection process, the global maximum within the maximal width of a heart sound [34] was detected, which could be located anywhere in that interval. Therefore, the distance between the first and the second heart sound could differ from the actual length of the corresponding systole. Therefore, a tolerance of 175 ms was applied on the length of a systole, which also took into account that the heart cycle can vary from one cycle to another. Thus, the tolerance of a systolic length was composed of the approx. maximal duration of a heart sound (150 ms) [34] and the standard deviation of 25 ms for a systolic length [35]. Hence, the minimal and maximal systolic length are given by

(8) $$\begin{array}{cc} {\mathrm{SYS}}_{\min} & =\mathrm{SYS}-175\;\mathrm{ms} \end{array}$$

(9) $$\begin{array}{cc} {\mathrm{SYS}}_{\max} & =\mathrm{SYS}+175\;\mathrm{ms}. \end{array}$$

The maximal and minimal length of a diastole are computed with

(10) $$\begin{array}{cc} {\mathrm{DIA}}_{\max} & =\frac{60\times 1000}{\mathrm{HR}}-{\mathrm{SYS}}_{\min} \end{array}$$

(11) $$\begin{array}{cc} {\mathrm{DIA}}_{\min} & =\frac{60\times 1000}{\mathrm{HR}}-{\mathrm{SYS}}_{\max}. \end{array}$$

### 3.5. Peak Classification

For the peak classification, two different approaches were developed for two different heart rate domains. At normal heart rates, the amplitude of S_1_ is not necessarily higher than that of S_2_ [40]. The amplitude of S_1_ increases approximately linear with the heart rate [41]. Thus, at increased heart rates, the amplitude of S_1_ is higher than that of S_2_ [42]. Therefore, at increased heart rates, the first and second heart sound can be distinguished based on their different amplitudes. Hence, at increased heart rates, noise and artefacts are negligible compared to the amplitudes of S_1_ and S_2_.

#### 3.5.1. Simple Heart Sound Classification for Increased Heart Rates (>80 bps)

**Figure 6:**
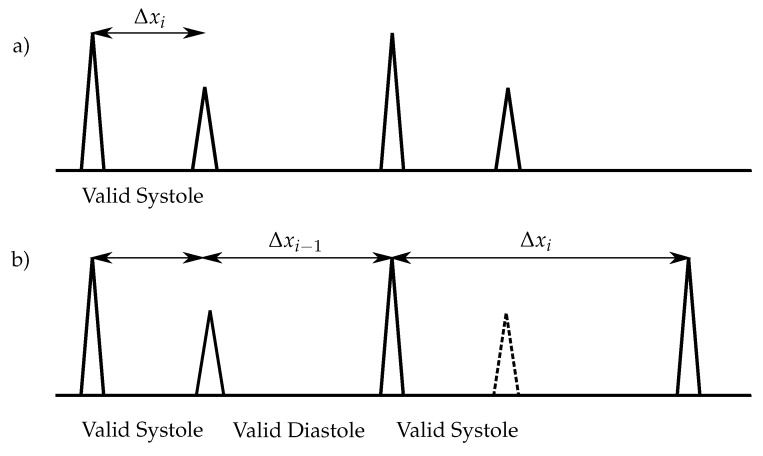
Heart sound classification for increased heart rates: The S_1_ have always higher amplitudes than the S_2_. (**a**) No S_2_ is missing; (**b**) One S_2_ is missing (dashed lines).

The peaks are classified into S_1_ and S_2_ by the condition

(12) $$\Delta x_{i}<{\mathrm{SYS}}_{\max}\;\wedge \;y_{i}>y_{i+1},$$

where $\Delta x_{i}$ is the *i*-th distance between two detected peaks and $y_{i}$ is the amplitude of the *i*-th peak. Therefore, the peak *i* is classified as S_1_ and i+1 as S_2_, respectively (Figure 6a). If one S_2_ is not detected, the following condition will classify the peak as S_1_ (Figure 6b):

(13) $$\Delta x_{i}>{\mathrm{SYS}}_{\max}\;$$

#### 3.5.2. Complex Heart Sound Classification for Normal Heart Rates (<80 bps)

For normal heart rates, the simple algorithm has to be extended by additional steps. It is necessary that peaks (e. g. caused by S_3_, S_4_ or artefacts), which would lead to wrong heart cycles, have to be neglected. Those extra peaks lead to invalid diastoles and have to be removed before the classification of the peaks. Therefore, the following condition is used:

(14) $${\mathrm{SYS}}_{\min}<\Delta x_{i-1}<{\mathrm{SYS}}_{\max}\;\wedge \;\Delta x_{i}<{\mathrm{DIA}}_{\min}\;\wedge \;{\mathrm{SYS}}_{\min}<\Delta x_{i+2}<{\mathrm{SYS}}_{\max}$$

**Figure 7:**
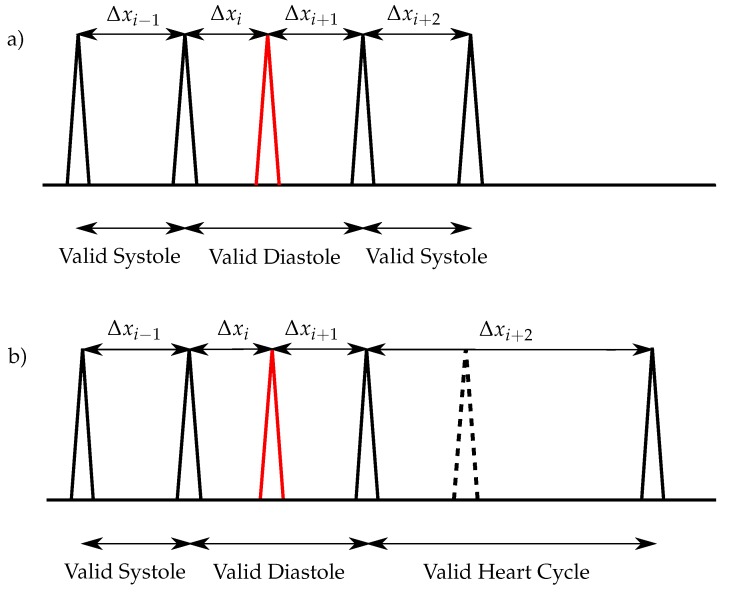
Heart sound classification for normal heart rates: (**a**) One extra sound (red) exists within the diastole and no S_2_ is missing; (**b**) One extra sound (red) exists within the diastole and one S_2_ is missing (dashed lines).

If this condition is fulfilled, the right peak of $\Delta x_{i}$ is neglected (Figure 7a). For the special case that no S_2_ is detected in $\Delta x_{i+2}$ and the condition

(15) $${\mathrm{DIA}}_{\min}<\Delta x_{i-2}<{\mathrm{DIA}}_{\max}\;\wedge \;{\mathrm{SYS}}_{\min}<\Delta x_{i-1}<{\mathrm{SYS}}_{\max}\;\wedge \;\Delta x_{i}<{\mathrm{DIA}}_{\min}$$

is true, the peak is removed as shown in Figure 7b.

**Figure 8:**
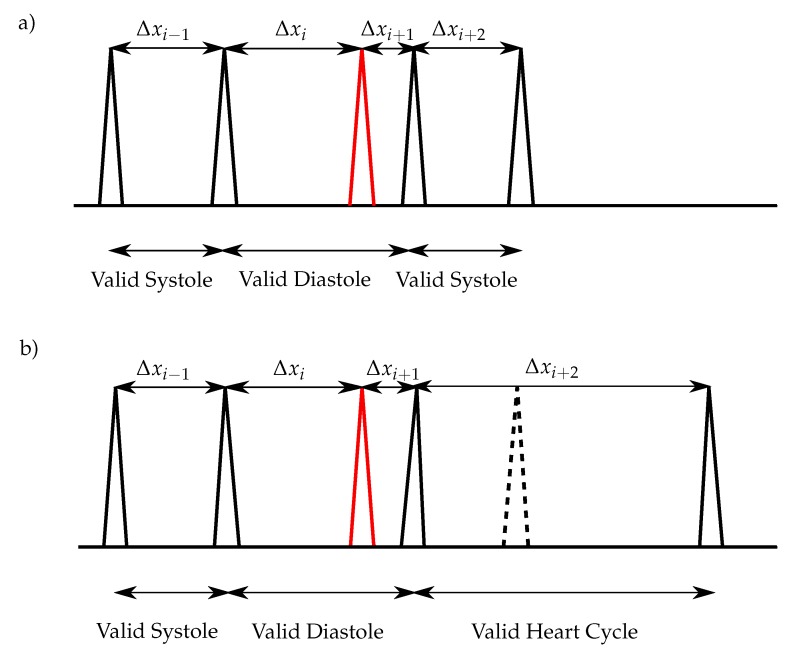
Heart sound classification for normal heart rates: (**a**) One extra sound (red), which is near to an S_1_, exists within the diastole and no S_2_ is missing; (**b**) One extra sound (red), which is near to an S_1_, exists within the diastole and one S_2_ is missing (dashed lines).

The peaks, which are removed by applying the aforementioned condition, lead to an invalid diastole length. However, if one extra-peak occurs shortly before S_1_, it can not be removed, since the corresponding length of the diastole is valid. Therefore, in the next step the remaining peaks between two valid systoles are removed with

(16) $${\mathrm{SYS}}_{\min}<\Delta x_{i-1}<{\mathrm{SYS}}_{\max}\;\wedge \;{\mathrm{DIA}}_{\min}<\Delta x_{i}<{\mathrm{DIA}}_{\max}\;\wedge \;{\mathrm{SYS}}_{\min}<\Delta x_{i+2}<{\mathrm{SYS}}_{\max}$$

(see Figure 8a). In the case that one S_2_ is missing and the following condition

(17) $${\mathrm{SYS}}_{\min}<\Delta x_{i-1}<{\mathrm{SYS}}_{\max}\;\wedge \;{\mathrm{DIA}}_{\min}\;<\Delta x_{i}<{\mathrm{DIA}}_{\max}\;\wedge \;{\mathrm{SYS}}_{\min}<\Delta x_{i+3}<{\mathrm{SYS}}_{\max}$$

is true, the right peak of the $\Delta x_{i}$ is removed (Figure 8b). In the case that two consecutive S_2_ are missing and the following condition

(18) $$\Delta x_{i}>{\mathrm{SYS}}_{\max}\;\wedge \;\Delta x_{i+1}>{\mathrm{SYS}}_{\max}$$

is true, the corresponding peaks are classified as S_1_. Due to the higher deviation of the diastolic length (heart rate variability), the algorithm performance is more stable by considering the systolic length for the aforementioned condition.

The remaining distances Δx are correct systoles, diastoles and heart cycles, therefore, with the information of the derived heart rate, systolic and diastolic length, the corresponding peaks can be classified into S_1_ and S_2_.

## 4. Statistical Evaluation and Optimization

For the first heart sounds, the peaks of the R-wave out of the ECG are used as a reference, whereas the classification of the second heart sounds is not evaluated with the ECG. Therefore, the performance of the classification algorithm is statistically evaluated in terms of the sensitivity, specificity, accuracy, precision and the F_1_-score only for S_1_. These parameters are defined as

(19) $$\begin{array}{cc} \mathrm{Sensitivity} & =\mathrm{Recall}=\frac{t_{p}}{t_{p}+f_{n}} \end{array}$$

(20) $$\begin{array}{cc} \mathrm{Specificity} & =\frac{t_{n}}{f_{p}+t_{n}} \end{array}$$

(21) $$\begin{array}{cc} \mathrm{Accuracy} & =\frac{t_{p}+t_{n}}{t_{p}+t_{n}+f_{p}+f_{n}} \end{array}$$

(22) $$\begin{array}{cc} \mathrm{Precision} & =\frac{t_{p}}{t_{p}+f_{p}} \end{array}$$

(23) $$\begin{array}{c} F_{1}\text{-score}=\frac{2\times \mathrm{Recall}\times \mathrm{Precision}}{\mathrm{Precision}+\mathrm{Recall}}. \end{array}$$

For evaluating the proposed algorithm, a tolerance window TW, which was applied around the peak of the R-wave, was introduced. The window was necessary, since the synchronization of the PCG and ECG was only an approximation and the maximum of a heart sound did not always occur at the beginning of the corresponding heart sound. Taking the maximal duration of a heart sound in consideration, a tolerance window of 150 ms was appropriate [34,43]. If a peak, which was classified as S_1_, lay within the window, the heart sound was correct. $f_{p}$ is the number of wrongly classified S_1_ peaks, which were outside of TW and $f_{n}$ are correct heart sounds, which were not detected by the algorithm. $t_{p}$ is the number of correctly classified S_1_ peaks and $t_{n}$ is the number of correctly as false classified S_1_ peaks.

All performance parameters were computed for all measurements for both, the HT and the STFT. The classification of the heart sounds was optimized to achieve the highest F_1_-score. In case of a high heart rate, the threshold *n* for peak detection was optimized separately. The results of the optimization process can be found in Section 6.1.

## 5. Data Acquisition

### 5.1. Measurement Devices and PC Setup

For recording the PCG, the electronic stethoscope 3M^TM^ Littmann^®^ model 3200 was used. The recorded data was sampled with 4 kHz. Chen et al. proposed that sampling rates above 5 kHz are not sufficient for heart sound recording, since for higher sampling rates, irrelevant sound components can be included [7]. The integrated microphone of the stethoscope amplifies frequencies between 20–200 Hz, since heart sounds are within this frequency range (see Section 2.1).

The classification of the first heart sounds was validated by an ECG. For this purpose a COR12 ECG device from Corscience was used, which has 12 channels and a sampling rate of 500 Hz. The classification algorithm was performed in MATLABmathsizesmall R2019a with a PC with an Intel^®^ Xeon^®^ E-2136 Processor at 3.3 GHz and 32 GB RAM. The computational time of the algorithm for the heart sound detection and classification was assessed for both, the HT and STFT.

### 5.2. Study Population and Protocol

For the study the PCG and ECG from 12 healthy male subjects, with no known heart diseases, were recorded. Their ages varied in a wide range between 24 and 68. An overview of the probands is given in Table 1. For every person, 10 different measurements were conducted. The duration of each measurement was 60 s. If nothing else was indicated, the measurement was conducted at Erb’s point while the test person was sitting. The different types of measurements are listed in Table 2. As the first measurement was conducted under optimal conditions it serves as reference. For the other measurements the posture, physical stress and auscultation position were varied.

## 6. Results and Discussion

### 6.1. Results of Optimization

The performance of the presented algorithm was optimized regarding the F_1_-score. The values of the optimized parameters are listed in Table 3. The threshold parameters *n*_normal_ and *n*_high_ were greater for the HT, since through the averaging effect of the STFT, its resulting envelope curve was smoother. The cut-off frequencies of the HT and STFT for the low-pass filter were 40 Hz and 20 Hz, respectively, and the cut-off frequencies of the HT and STFT for the high-pass filter were 190 Hz and 120 Hz, respectively. The filter for both, the HT and STFT, were from the order of 10 for the low-pass filter and 4 for the high-pass, respectively.

The filter suppressed noise, which was caused by human voice, respiration or lung sounds. The fundamental frequency of human voice is approximately 120 Hz for male and 190 Hz for female, respectively [44]. Therefore, the applied filter eliminated the majority of human voices. However, no study about the influence of speaking during the measurements was made. The frequency range of lung sounds and respiration is approximately 60–1200 Hz [45,46]. Thus, lung sounds and breathing were partly suppressed by the filtering. However, artefacts from the lung could not be fully eliminated, since heart sounds occurred within that frequency range. The cut-off frequencies were the result of the optimization process, regarding the average F_1_-score.

### 6.2. Comparison of the Two Approaches for Systolic Length Estimation

**Figure 9:**
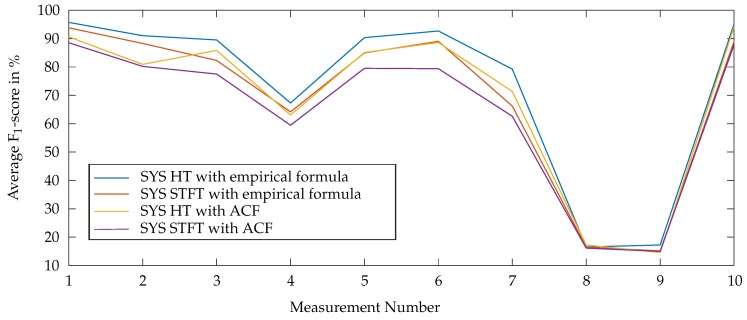
Comparison of the ACF-based and the empirical-based method for the systolic length estimation: the average F_1_-score is plotted over the ten conducted measurements.

As introduced in Section 3, two different methods were considered for the systolic length extraction: based on the ACF and based on the empirical formula 6. For each of these methods and for both, the HT- and STFT-based approach, F_1_-scores were calculated. The results for these combinations are shown in Figure 9. The proposed algorithm achieves a better performance by using the empirical formula for the systolic length estimation. Therefore, in the following the algorithm is evaluated by using the empirical approach.

### 6.3. Results of Heart Sound Classification

The respective average values of the performance parameters are listed in Table 4. Furthermore, the average performance parameters were calculated without the measurements 4, 8 and 9, since the reference ECG was noisy for the measurement 4 and the measurements at the back (8 and 9) had a noisy PCG. The evaluation results for the F_1_-score for the single probands and measurements are shown in Table 5 and Table 6. Measurement 1 was the reference for the other measurements. It showd the best results, since it was conducted under optimal circumstances at Erb’s point.

### 6.4. Influence of Different Measurement Conditions

#### 6.4.1. Varying the Auscultation Point

The measurements 1, 7, 8 and 9 were conducted during a sitting position and in the resting state. Only the auscultation point was varied. As the results in Table 4, Table 5 and Table 6 suggest, the F_1_-score was best for Erb’s point (Measurement 1) as expected, whereas measurement 7 was performed at the sternum. The average F_1_-score was lower compared to the reference measurement 1 and varied largely between the single subjects. The reason for that performance is the lower amplitude of the heart sounds (see Section 2.1). Therefore, the signal-to-noise ratio suffered and noise could be misinterpreted as heart sounds.

The results for measurements 8 and 9, which were performed on the back of the subjects, provided poor results for heart sound classification. The reason for that is the weak acoustic signal, which is attenuated by the lunges and the backbone. In consequence, it is not advisable to place a wearable system for heart sound monitoring on the back, as suggested in [47]. Therefore, the average of the evaluation parameters was calculated without the measurements 8 and 9 as well.

#### 6.4.2. Varying the Posture

The measurements 1, 3, 4, 5 and 6 were conducted with different postures of the probands. Measurement 3, 5 and 6, where the probands were lying on the back, lying on the right side and lying on the left side, showed similar values regarding the performance parameters. However, compared to measurement 1, the results were slightly worse.

The results for measurement 4 were poor, since during the measurement the subjects were lying on the stomach. This led in some cases to noise in the ECG, which was caused by movement of the electrodes. In consequence, the reference signal was distorted and the evaluation of the classification performance suffered. However, the PCG was not affected.

#### 6.4.3. Varying the Physical Stress

Measurement 2 was conducted with deep breathing and measurement 10 after 5 minutes of sport, respectively. This reflects physical stress situations. The results of measurement 10 show that the classification of heart sounds worked well for increased heart rates. The average ratio of the amplitudes of S_1_ and S_2_ for increased heart rates was 1.8 for the STFT and 3.4 for the HT. Therefore, S_1_ could be distinguished easily from noise as well as from S_2_. Moreover, the results of measurement 2 showed that deep breathing hardly affected the classification algorithm.

#### 6.4.4. Influence of BMI

The probands were arranged according to their BMI and, therefore, divided into two groups of equal size. Group “low BMI” consists of proband 1, 3, 4, 6, 8 and 10 and group “high BMI” of 2, 5, 7, 9, 11 and 12. The average F_1_-score without the measurements 4, 8 and 9 was computed for both the HT and STFT and compared for both groups (see Table 7). The results for the group “high BMI” showed that the average F_1_-score was approximately 4% worse than the group “low BMI”, regarding for both, the HT and STFT, since the heart sounds were more attenuated for higher BMIs. In consequence, the algorithm was quite robust towards a variation of the BMI, for envelope extraction for both the HT and STFT.

### 6.5. Comparison of HT and STFT

Overall, the average F_1_-score by using the HT for extracting the envelope curve was approx. 5% better than those with the STFT. Due to the fact that the STFT was computed within a time window, the time resolution was limited, since an appropriate frequency resolution was needed. In consequence, the number of samples was reduced and, therefore, the accuracy of the derived length of the systoles was smaller, resulting in incorrectly removed S_1_. This effect was even increased in case of split S_1_.

**Figure 10:**
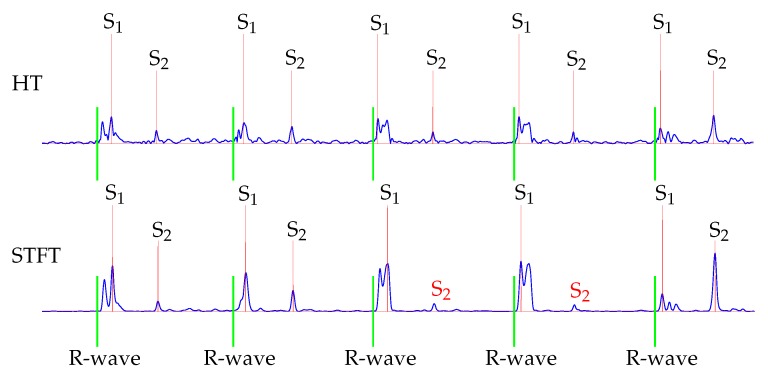
Example of classified heart sounds, where the detection of S_2_ failed in some cases for the STFT (red). The reference ECG is shown in green.

Furthermore, the classification for S_2_ showed that the HT performed better. This is because the STFT was computed within a time interval, which led to an averaging of the amplitudes. Therefore, in some cases the maximal power spectral density was reduced, which was used as the envelope curve for the classification. An exemplary issue is shown in Figure 10. The fourth and fifth S_2_ were not detected by using the STFT for envelope curve extraction.

Regarding the goal of a wearable sensor solution for daily health monitoring, the computational cost and time were essential. A comparison between the computational effort of the HT and STFT is given in Table 8. This means that a 60 s PCG signal was classified within approximately 140 ms for the HT and 480 ms for the STFT, respectively. In consequence, both methods can be regarded as real-time capable, but nevertheless, the algorithm based on the HT performed about 3 times faster than the STFT.

Wearable systems have limited computational capacity as well as power supply. Thus, it is essential to use a computational low complex algorithm for the real-time monitoring of daily life activities. Hence, the HT can be ranked as more appropriate for this purpose compared to the STFT approach.

### 6.6. Comparison with other Approaches

In the following, the performance parameters for the S_1_ classification of the proposed algorithm are compared to other algorithms for the heart sound classification (see Table 9). As aforementioned in Section 1, there are three well-established groups of algorithms for heart sound classification: the feature-based, probabilistic-based and envelope-based methods. Therefore, the performance of algorithms, which represent the state of the art, was compared to the presented algorithm ones. However, it has to be noted that the performance parameters could not be directly compared to each other, since the proposed data set differed from the others. Therefore, the reference measurement of the proposed data set was used, since it was conducted under optimal conditions like it is normally applied in the literature. Furthermore, no standards for measurements and evaluation of the algorithms exist, which led to non-uniform performance parameters. Since the performance of the presented algorithm is best by using the HT, it was used for the comparison.

The database size in the literature is in most cases very small compared to the proposed one (60×120 s). Only Springer et al. used a larger database than the proposed one [12]. Furthermore, in many approaches very short recordings are included in their database, for example ~1 s by Renna et al. [14], or in total 87 heart sounds by Chen et al. [7]. Moreover, many researchers use a database like PhysioNet and do not declare their study population or recording length [6,9,39]. Other researchers have conducted their measurements under optimal conditions (apart from [39]) and no variation of the posture, auscultation point, physical stress and breathing was considered within their studies. Furthermore, the proposed study population includes only healthy subjects, in [16,18,39] this was also the case.

The feature-based methods have the best performance parameters [6,9]. However, since feature- based methods have a strong dependency on their training datasets and a high computational effort, they are not the favourable methods for a low complex wearable sensor platform to monitor daily activities in real-time. This holds also true for probabilistic-based methods presented in [12,14]. However, in [39] a real-time capable probabilistic-based method was realized with a low-cost smartphone platform. The performance, however, is poorer as the state of the art suggests, including the proposed one.

Even the average performance for different physiological conditions (e.g., physical stress, posture, BMI, auscultation point, breathing) of the presented algorithm is quite good compared with the state of the art. In consequence, the developed algorithm is robust and appropriate for a wearable sensor platform.

The presented algorithm is not able to deal with more than one extra peak, nor is able to classify pathological sounds (e.g., murmur). Thus, the performance suffers, if more than one detected peak exists within a diastole. For the probabilistic-based methods even one extra peak can be problematic, since it can lead to wrong states in their sequence.

## 7. Conclusions

This paper presents an enveloped-based and real-time capable algorithm for the detection and classification of the heart sounds S_1_ and S_2_ in phonocardiograms (PCG). The peaks of the envelope curve were classified and the found S_1_ were compared to the reference ECG. The algorithm was tested using the Hilbert transform (HT) and short-time Fourier transform (STFT) as methods to extract the envelope curve out of the PCG. The results for the heart sound classification suggested that using the HT is more favourable, due to the better performance parameters and lower computational effort. The developed algorithm is robust against the variation of the posture, heart rate, BMI, age and auscultation point, except for the back, since the PCG signals are attenuated by the lungs and backbone. As expected, the auscultation at Erb’s point provides the best result followed by the sternum. The posture and physical effort hardly effect the performance of the proposed algorithm for heart sound classification. Furthermore, the algorithm is adapted in order to deal with additional peaks caused by noise and an equal length of the systole and the diastole by an increased heart rate, respectively. Thus, the proposed measurements reflect and predict daily situations of the probands.

In the future, the envelope curves of the HT and STFT will be combined in order to increase the accuracy of the classification, since both envelope curves contain different information. Moreover, the heart rate could be estimated with the non-negative matrix factorization (NMF) out of the spectrogram, as suggested by [23]. Therefore, the algorithm with the STFT approach could be improved. Furthermore, the presented algorithm will be combined with activity classification, as proposed in [4]. For this purpose, the computational effort of the proposed algorithm must be reduced by an optimization of the implemented code as well as a reduction of the sampling rate of the PCG.

## Figures and Tables

**Table 1:** Overview of the subjects.

Proband No.	Age in a	Weight in kg	Body Height in m	BMI in kg/m${}^{2}$
1 (reference)	26	78	1.80	24.1
2	68	92	1.78	29.0
3	28	82	1.85	24.0
4	27	79	1.88	22.4
5	24	75	1.72	25.4
6	26	68	1.78	21.5
7	29	82	1.82	24.8
8	37	75	1.80	23.1
9	55	87	1.74	28.7
10	38	72	1.91	19.7
11	34	80	1.71	27.4
12	28	80	1.83	24.2
**Average**	**35**			**24.9**

**Table 2:** Study protocol.

Meas. No.	Action	Breathing	Posture	Auscultation Point
1	rest	normal	sitting	Erb’s point
2	rest	deep	sitting	Erb’s point
3	rest	normal	lying on back	Erb’s point
4	rest	normal	lying on stomach	Erb’s point
5	rest	normal	lying on right side	Erb’s point
6	rest	normal	lying on left side	Erb’s point
7	rest	normal	sitting	sternum
8	rest	normal	sitting	centre of the back
9	rest	normal	sitting	left side of the back
10	after 5 min of sport	deep	sitting	Erb’s point

**Table 3:** Optimization results for of the heart sound classification.

	Parameter	STFT	HT
Threshold parameter normal heart rate	*n*_normal_	1	1.9
Threshold parameter increased heart rate	*n*_high_	0.6	1.3
Lower cut-off frequency in Hz	$f_{\mathrm{lower}}$	20	40
Upper cut-off frequency in Hz	$f_{\mathrm{upper}}$	120	190
Filter order high-pass	$N_{\mathrm{HP}}$	4	4
Filter order low-pass	$N_{\mathrm{LP}}$	10	10
Window-size STFT in Samples	*b*	128	-
Overlap ratio STFT in %	*k*	96.875	-

**Table 4:** Average values of the evaluation parameters (in %) for the single measurements.

	**ø Sensitivity**		**ø Specificity**		**ø Accuracy**		**ø Precision**		**ø F_1_-Score**	
**Measurement No.**	**HT**	**STFT**	**HT**	**STFT**	**HT**	**STFT**	**HT**	**STFT**	**HT**	**STFT**
1 (reference)	95.1	92.5	99.2	99.0	98.5	97.9	96.5	95.3	95.7	93.8
2	88.8	85.5	98.8	98.4	97.1	96.2	93.7	91.5	91.0	88.3
3	87.0	80.0	98.7	97.3	96.7	94.5	92.5	84.9	89.5	82.3
4	62.5	59.8	95.5	94.8	85.0	83.9	78.4	74.9	67.3	64.2
5	88.9	83.0	98.4	97.8	96.9	95.4	92.0	88.7	90.3	84.9
6	90.9	87.3	99.0	98.3	97.6	96.6	94.6	91.0	92.7	89.0
7	76.5	65.2	96.7	94.4	93.3	89.4	82.4	68.9	79.2	66.2
8	17.6	18.6	81.8	81.6	71.6	71.5	16.3	16.9	16.5	16.5
9	18.6	15.9	81.2	85.0	71.1	73.9	17.9	19.0	17.2	15.2
10	95.9	86.7	98.4	98.5	97.9	95.9	94.6	94.1	95.2	89.0
**ø**	**72.2**	**67.5**	**94.8**	**94.5**	**90.6**	**89.5**	**75.9**	**72.5**	**73.5**	**68.9**
**ø without 4, 8 and 9**	**89.0**	**82.9**	**98.5**	**97.7**	**96.9**	**95.1**	**92.3**	**87.8**	**90.5**	**84.8**

**Table 5:** F_1_-score (in %) of the classified PCG by using the HT.

**Proband No.**	**1**	**2**	**3**	**4**	**5**	**6**	**7**	**8**	**9**	**10**	**11**	**12**
Meas. No. 1	94.6	100.0	96.8	92.9	94.4	96.2	100.0	98.3	96.9	82.8	95.8	100.0
2	92.3	99.2	97.4	90.6	96.6	92.3	74.1	92.7	98.1	90.9	73.0	95.2
3	91.1	89.5	100.0	74.2	86.3	87.5	95.4	91.7	77.7	94.6	94.0	92.4
4	50.0	78.0	95.4	69.1	70.1	20.8	80.0	24.8	96.1	93.5	41.2	88.5
5	87.3	94.0	91.3	94.8	84.9	96.4	99.1	86.5	91.4	97.5	81.6	79.3
6	95.2	88.3	96.0	95.1	80.0	95.6	95.9	84.2	96.6	99.2	94.3	91.8
7	85.1	70.0	97.0	82.8	97.3	81.7	76.6	89.7	49.6	86.4	79.6	54.9
8	15.2	14.0	10.4	15.2	12.7	13.5	30.5	15.9	28.6	19.6	15.3	7.69
9	9.76	15.5	11.6	14.4	16.8	10.5	3.42	25.2	19.0	41.4	20.5	18.8
10	100.0	94.3	98.0	91.4	96.4	99.4	96.2	97.9	86.9	97.6	86.2	97.8
**ø**	**72.1**	**74.3**	**79.4**	**72.1**	**73.5**	**69.4**	**75.1**	**70.7**	**74.1**	**80.4**	**68.2**	**72.6**
**ø without 4, 8 and 9**	**92.2**	**90.8**	**96.6**	**88.9**	**90.8**	**92.7**	**91.0**	**91.6**	**85.3**	**92.7**	**86.4**	**87.4**

**Table 6:** F_1_-score (in %) of the classified PCG by using the STFT.

**Proband No.**	**1**	**2**	**3**	**4**	**5**	**6**	**7**	**8**	**9**	**10**	**11**	**12**
Meas. No. 1	87.5	100.0	95.2	89.1	87.4	92.1	100.0	97.4	93.8	85.7	97.5	100.0
2	95.1	96.1	93.9	92.5	90.4	93.3	60.4	92.7	96.3	82.8	75.9	90.2
3	91.1	94.8	100.0	57.1	71.0	82.2	87.0	43.8	84.2	90.1	94.0	92.4
4	49.3	82.3	95.4	74.3	68.1	15.4	83.6	20.4	89.0	96.8	28.0	67.3
5	60.7	72.2	76.2	91.7	88.7	92.0	99.1	86.5	85.5	96.7	85.1	85.0
6	94.2	82.2	81.2	95.1	57.9	96.6	96.7	87.0	95.5	99.2	87.6	95.2
7	85.1	63.8	96.0	68.0	94.5	56.5	87.6	65.5	19.6	73.1	47.5	37.3
8	15.5	5.97	15.8	17.6	15.9	11.4	20.0	21.9	32.9	23.7	9.62	7.59
9	11.3	17.7	3.33	20.4	17.5	6.06	8.0	3.39	32.2	24.5	23.4	14.0
10	98.9	94.9	99.0	80.0	60.7	100.0	95.1	97.9	86.8	95.9	63.6	95.7
**ø**	**68.9**	**71.0**	**75.6**	**68.6**	**65.2**	**64.6**	**73.8**	**61.7**	**71.6**	**76.3**	**61.2**	**68.5**
**ø without 4, 8 and 9**	**87.5**	**86.3**	**91.6**	**81.8**	**78.7**	**87.5**	**89.4**	**81.5**	**80.2**	**89.1**	**78.7**	**85.1**

**Table 7:** Comparison of the average F_1_-score (in %) of the two groups with low and high BMI.

Group:	low BMI	high BMI
HT	92.5	88.6
STFT	87.5	83.1

**Table 8:** Comparison of the computational effort for Hilbert transform (HT) and STFT.

Computational	for All	Average for
Time	Measurements	60 s Measurement
HT	16.8 s	140 ms
STFT	57.6 s	480 ms

**Table 9:** Comparing the performance of the proposed algorithm with the state of the art heart sound classification algorithms. The performance parameters S, P, Acc and F_1_ correspond to the sensitivity, precision, accuracy and F_1_-score, respectively. The performance parameters are only related to S_1_, if not stated otherwise in the notes.

Reference	Year	Database	Study	Performance in %				Notes
(Methods)			Population	S	P	Acc	F_1_	
**Envelope-based:**								
	2020							real-time capable
Proposed		120 meas.	12 healthy	**95.1**	**96.5**	**98.5**	**95.7**	reference
Algo. (HT)		à 60 s	subjects	(89.0)	(92.3)	(96.9)	(90.5)	(average of meas. with
								diff. physio. conditions)
[18]	2014	600 s total	45 healthy	–	–	98.5	–	short records
(STMHT)			subjects					
[16]	2014	80 records	6 healthy	96	95	–	–	meas. in acoustic
(S-Transform)		à 6–12 s	subjects					chamber, short records
**Probabilistic-based:**								
[12]	2016	10,172 s	112	–	–	–	97.0	healthy and path.
(HSMM)		total	subjects					subjects, large database
[39]	2018	–	15 healthy	–	–	76.9	–	real-time capable,
(DWT+CWT								smartphone platform,
+HMM)			subjects					parameters for S_1_ and S_2_
[14]	2019	1–35 s	135	95.7	95.7	93.7	–	healthy and path. subj.,
(CNN+HMM)		records	subjects					parameters for S_1_ and S_2_
**Feature-based:**								
[7]	2017	87	6	–	–	91.1	–	small study population,
(DNN		heart	subjects					short records,
+MFCC)		sounds	(test)					parameters for S_1_ and S_2_
[9]	2018	1000 records	–	98.2	–	97.9	99.7	healthy and path. subj.,
(SVM+MFCC								unspecif. study pop.,
+DWT)								parameters for S_1_ and S_2_
[6]	2019	409	–	98.6	99.4	98.6	99.0	healthy and path. subj.,
(MDS		records						unspecif. study pop.,
+TWSVM)		(PhysioNet)						parameters for S_1_ and S_2_

## Supplementary Materials

The following are available online at https://www.mdpi.com/1424-8220/20/4/972/s1.

## Abbreviations

The following abbreviations are used in this manuscript:

ACF	Autocorrelation function
BMI	Body-mass index
CNN	Convolutional neural network
CWT	Continuous wavelet transform
DNN	Deep neural network
DWT	Discrete wavelet transform
ECG	Electrocardiogram
FT	Fourier transform
HMM	Hidden-Markov model
HSMM	Hidden-semi Markov model
HT	Hilbert transform
MDS	Multidimensional scaling
MFCC	Mel-frequency cepstral coefficients
NMF	Non-negative matrix factorization
PCG	Phonocardiogram
S_*i*_	*i*-th heart sound
STFT	Short-time Fourier transform
STMHT	Short-time modified Hilbert transform
SVM	Support vector machine
TWSVM	Twin support vector machine
